# Adaptability in healthcare buildings: a perspective through Joseph Bracops Hospital

**DOI:** 10.3389/fmedt.2023.1199581

**Published:** 2023-07-10

**Authors:** Giulia Scialpi, Joost Declercq

**Affiliations:** ^1^ Archipelago Architects, Brussels, Belgium; ^2^ Urban Metabolism Lab, Louvain Research Institute for Landscape, Architecture, Built Environment, Université Catholique de Louvain, Brussels, Belgium

**Keywords:** adaptability assessment model, flexibility, adaptability, healthcare buildings, building obsolescence

## Abstract

The healthcare sector has to face changes happening fast and often in an unpredictable way, such as epidemiological trends, the advancements of medical technology and processes or evolving social and economic needs. This results in a frequent need for infrastructures' retrofitting, with an increasing focus on the environmental impact of buildings, which have one of the highest embodied carbon footprints per square meter in the construction sector. As result, interest in healthcare buildings' adaptability is growing among researchers and practitioners. After an introduction on the research topic, a focus on the definition of adaptability and the existing assessment models is provided to address the following research question: to what extent are adaptability models effective to evaluate and orient the design of healthcare buildings? A quite varied use of the term adaptability has been found in the literature, as well as a new research trend aiming to establish a link with circularity. Moreover, most of the assessment models do not have a focus and have never been tested on the healthcare sector. An approach to circular and adaptable design is presented through the case study of the Joseph Bracops Hospital (Belgium), which has been submitted for evaluation by the Reversible Building Design protocol developed by Dr. Durmisevic. The evaluation highlights some of the current barriers in the design of adaptable healthcare facilities. Insights for future research are provided to encourage data-collection about the service life of healthcare buildings, so to understand if the adaptability of these infrastructures should be mainly monofuntional or transfunctional.

## 1. Introduction

In the last decades, healthcare facility obsolescence has accelerated, resulting in a shorter lifecycle for infrastructures that need to be changed before the end of their physical lifecycle (1). In the case of hospitals, this may be due to: technological advancements; the long duration of the project conception and construction, leading to the delivery of an already inadequate infrastructure (2); the uniqueness of their architecture that makes it difficult to foresee a routineness in retrofit projects (3), or societal changes and crisis such as the coronavirus which has revealed the limits of healthcare buildings facing unforeseen situations (4). This results in a frequent need to retrofit healthcare infrastructure, creating a paradox by which healthcare facilities are considered as an energy-intensive building type that contributes substantially to environmental impacts while accidentally leading to diseases and adverse health outcomes (5). According to Health Care Without Harm Europe, if the healthcare sector was a country, it would be the fifth largest emitter in the world, with a climate footprint equivalent to 4.4% of global net emissions (6).

Investing in zero emission buildings and infrastructure is the most powerful action to reduce carbon emissions towards a zero carbon healthcare sector by 2050 (6). Reducing energy consumption is essential (7) but additionally, since healthcare infrastructure buildings have the highest mean embodied carbon (EC) per m^2^ amongst all studied building types with values around 800 kg CO_2_e/m^2^ (8), a triple design approach can be undertaken. First, every effort should be made in the planning, design, and refurbishment of facilities to ensure that space utilization is maximized and only absolutely necessary buildings are constructed (6). Second, the environmental life cycle impact of the used materials and technical installations should be as low as possible. Third, the flows of the materials involved should be optimized according to circular design principles (9). In this context, the adaptability of healthcare buildings is gaining interest among practitioners and academics.

In this paper, the concept of adaptability is presented through a brief state of the art to clarify its definition and the assessment models available with a focus on healthcare facilities such as hospitals. The case study of Joseph Bracops Hospital is presented to show an approach to circular and adaptable building design and to help address the following research question: *to what extent are adaptability assessment models effective to evaluate and orient healthcare building design*?

## 2. Adaptability in healthcare buildings

The inconsistent use of the terms *adaptability* or *flexibility* of buildings has been widely reported in the literature by (1, 3, 10, 11) among others, which aim to clarify those concepts. In these studies, the concept of adaptability differs from flexibility, elasticity or reversibility. Adaptability is generally considered as the capacity of a building to accommodate change (3). Arge introduces an interesting classification, according to which the adaptability concept should be divided into three levels of increasing importance: generality (regarding changes that require minimal intervention inside the spaces); flexibility (including heavy modifications such as the conversion of spaces) and finally elasticity (referring to major structural changes which allow the building to expand in an horizontal and vertical way) (2). Moreover, not only the effort spent for the adaptation should be considered but also its timeframe (12, 13). The high change rate in programmatic needs means that several building elements have a lifecycle significantly shorter than their potential technical lifespan. Hence, three system levels coupled with adaptability degrees and different lifespans are distinguished in a hospital: the tertiary system (furniture, medical equipment etc.) with a 5–10 year lifecycle; the secondary system (partition walls, building services) with a 20–50 year lifecycle; and the primary system (bearing structure) with a 100 year lifecycle. Based on this distinction, we can consider the secondary system as one of the most crucial because it straddles two types of planning horizons: a short to midterm planning based on statistical analysis and emerging models of care, and a mid- to longer-term based on much less firm knowledge and a more general hypothesis (14). There is a limited amount of literature about healthcare building adaptability and a lack of models to assess it (1, 4). While other typologies of buildings such as residential, offices, or commercial buildings are designed to be loose fit, hospitals need to be designed to optimally fit a specific function and still be able to deal with uncertainties. Only a few studies tackle the question whether it is more efficient to design for an unknown future function or for a specific function that could hardly host changes.

### 2.1. Brief state of the art on building adaptability and its assessment models

The growing interest in building adaptability has been reviewed by Heidrich et al., who provided an overview of the research works conducted between 1990 and 2017 focusing on the meaning of building adaptability and its diverse uses, on a list of the different adaptability strategies and a review of theories and models (15); by Pinder et al., who explored the controversial definition of adaptability from the practitioners' point of view through a set of interviews with architects, engineers, developers etc. (10); by Rockow et al., who realized a comprehensive review of available models (11); by Askar et al., who highlighted the link between the concept of Design for Adaptability (DfA) and circular building strategies through an analysis of adaptability and circularity assessment models (16); and finally by Hamida et al., who conducted an integrative literature review to understand circular building adaptability (17).

These research works provide an overview of the existing body of literature. Based on their findings a new research trend that aims to clarify the link between circularity and adaptability in buildings is emerging, since the concept of DfA can be considered as a powerful enabler to the circular building strategies (18). Adaptability and circularity are both aimed to improve the building lifecycle and require dynamics in building configuration and composition, however while “adaptability” focuses on facilitating building alteration, “circularity” focuses on achieving efficient flows of building assets (17). This synergy is particularly interesting when thinking to the European Policies and goals about the circular economy in the construction sector (19) and its nexus with the achievement of the United Nations' Sustainable Development Goals, impulsed by the introduction and approval of the UN 2030 agenda (20). The Reversible Design Protocol, developed by Dr. E. Durmisevic, tries to establish this connection between adaptability and circular design (21). The model, which is presented in the next paragraph through the Joseph Bracops Hospital case study, aims to integrate the design for adaptability and disassembly with the reuse and recycling of materials.

Moreover, there is a consensus about the scarcity of data-driven and empirically validated assessment models (11, 15–17). A summary of the existing models is presented in Table 1. The models are organized in a chronological way, specifying whether they are applicable for new or existing buildings according to the functions considered and according to the main factors of building obsolescence that depend on a wide range of parameters (25, 34):

1. Physical (structural failure, physical deterioration etc.);
2. Economic (changing demand for the goods or services, site's features, transport facilities etc.);
3. Functional (structural grid, free height, disassembly potential etc.);
4. Technological (orientation, insulation, natural lighting etc.);
5. Social (history, aesthetics etc.);
6. Legal (safety, security, disability access etc.)
7. Political (ecological footprint, masterplan, community support etc.).

**Table 1:** Summary of adaptability assessment models. Adapted from (11, 16).

Model	Author	Year	Factors	Domain	Function
Adaptive Reuse Potential (ARP)	Langston and Shen (22)	2007	✓ physical ✓ economic ✓ functional ✓ technological ✓ social ✓ legal	Existing	General
The Adaptable Building Design (ABD) framework	Allahaim, Anas, and Leifer (23)	2010	✓ economic ✓ technological ✓ legal	New	Office/commercial
IconCUR	Langston and Smith (24)	2012	✓ physical ✓ technological ✓ economic ✓ social ✓ legal ✓ political	Existing	General
AdaptSTAR	Conejos, Langston, and Smith (25)	2013	✓ physical ✓ functional ✓ technological ✓ economic ✓ social ✓ legal ✓ political	Existing/New	General
Causal Loop Diagram of building adaptation (CLD)	Gosling et al. (26)	2013	✓ physical ✓ functional ✓ technological ✓ economic ✓ political ✓ social ✓ legal	Existing	General
Preliminary Assessment Adaptation Model (PAAM)	Wilkinson (27)	2014	✓ functional ✓ technological ✓ economic ✓ social ✓ legal ✓ political	Existing	Office/commercial
FLEX 4.0.	Geraedts (28)	2016	✓ technological ✓ functional ✓ legal ✓ economic	Existing	General/office/school
Triple-bottom-line retrofit optimization	McArthur and Jofeh (29)	2016	✓ technological ✓ economic ✓ social	Existing	General
Learning Building Framework (LBF)	Ross (30)	2017	✓ functional	New	General
Conversion Meter	R. P. Geraedts, van der Voordt, and Remøy (31)	2017	✓ physical ✓ functional ✓ technological ✓ economic ✓ legal ✓ social ✓ political	Existing	Office/housing
Transformation Capacity (TC)	Durmisevic (21)	2018	✓ functional ✓ technological ✓ legal	Existing/new	Office/school/housing
Spatial Assessment of Generality and Adaptability (SAGA)	Herthogs et al. (32)	2019	✓ functional	Existing/new	Housing
Adaptive Reuse Assessment Model (ARAM)	Mehr and Wilkinson (33)	2021	✓ physical ✓ technological ✓ economic ✓ social ✓ legal ✓ political	Existing	Heritage

As Table 1 indicates, there is currently no model focusing on healthcare. The healthcare building adaptability studies found in the literature are mainly related to design principles such as the Open Building Design. This concept was developed for residential architecture in 1961 by Habraken (35) and then used as an assessment model for healthcare facilities by Capolongo et al. (36) and with some modifications by Brambilla et al. (4). In the related Open Building Assessment tool (OBAT) used by Capolongo et al., a case study can be evaluated according to eight parameters: 1. shape, 2. structure, 3. façade, 4. building plan, 5. expandability, 6. restrictions, 7. technologies and 8. exchangeability of large equipment (36). Each parameter is rated with a score between 0 and 10 points. The modified OBAT resulted in a version called Optimized Flexibility Assessment Tool (OFAT), “providing a critical review of the eight parameters and introducing a ninth one: functionality”. Functionality is an essential principle that considers efficiency and future-proofing design on six measurable variables (generic/universal rooms, space standardization, overflow design, loose fit, furniture/equipment flexibility, double function) (4). Kyrö et al. discuss some empirical interviews findings to suggest specific adaptability strategies in hospital retrofits, such as: standardization, multifunctional use, rooms conversion, empty chair and soft space concepts, interstitial floor, isolation, site repurposing and extensibility (3). An important part of their work lies in basing the adaptability indicators on different stakeholders' point of views: the medical staff, the patients, the designers, and the managers. The users' perspective, in particular that of the medical staff, has been extensively explored by the work of Durosaiye et al. (37) and Chrysikou et al. (38) who base their research on post-occupancy evaluation and feedback to feed and enhance healthcare facilities design. The results of this kind of research, providing qualitative and evidence-based data, should be integrated into adaptability frameworks.

### 2.2. The reversible building design protocol (RBD)

The Reversible Building Design (RBD) protocol was developed by Dr. Elma Durmisevic of TU Twente University/4D Architects in the framework of the Horizon 2020 research project BAMB2020. The RBD protocol focuses on two main aspects: the ability of a building to adapt to different functions during its lifetime (adaptability) and the ability to extract and exchange materials from the building without the need for major construction works (circularity potential) (21). The analyzed aspects are grouped under two main indicators: the Transformation Capacity (TC) and the Reuse Potential (RP) (39). The TC evaluates the ability of a building to host different functions all along its lifecycle, while RP measures the capacity of an assembly to be disassembled simply and without damages and thus ready to be reused. For the purpose of this research only the TC will be discussed.

**Figure 1:**
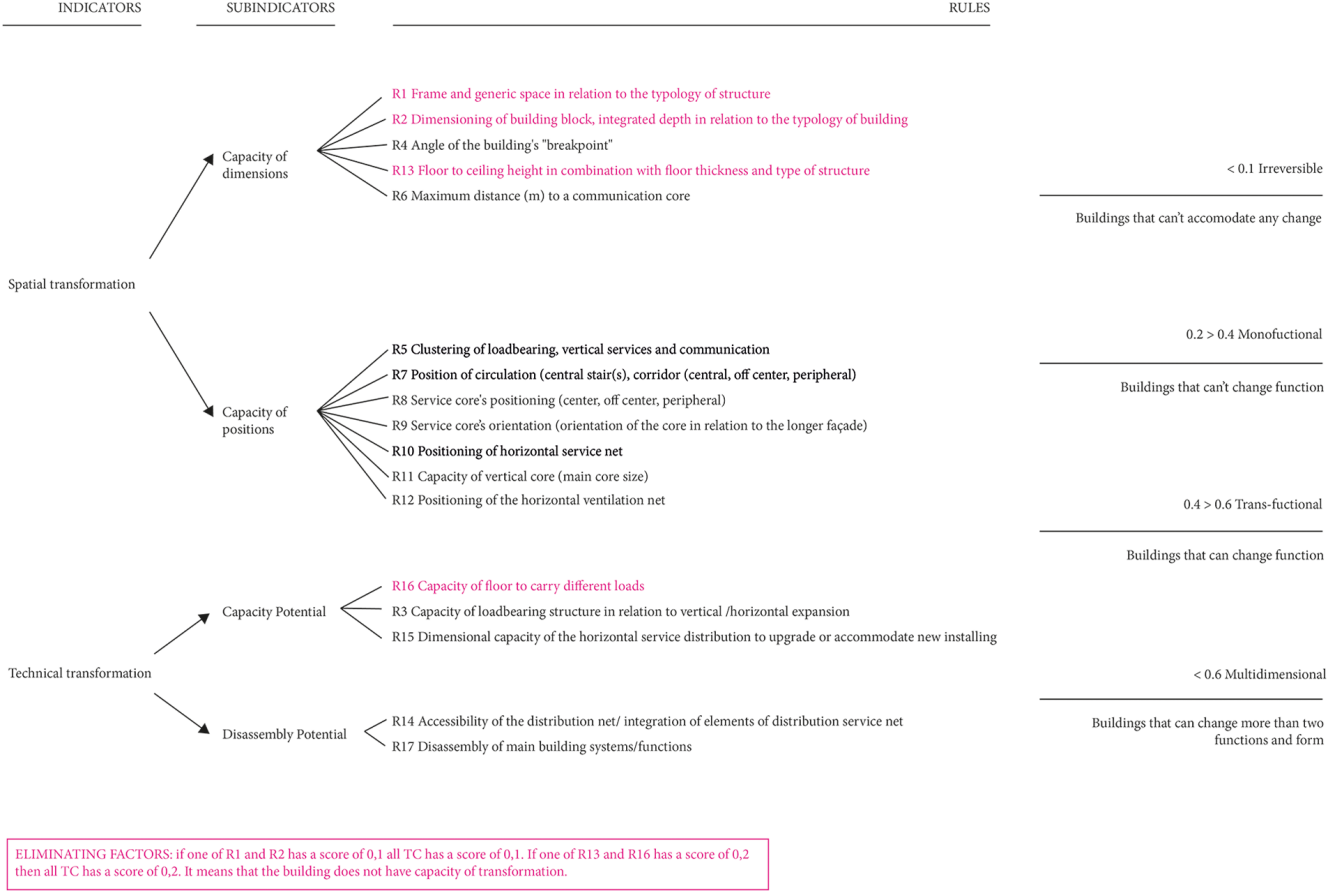
Scheme of Transformation Capacity (TC).

TC is based on four sub-indicators (Figure 1): (1) capacity of dimensions, which includes all the characteristics related to the building block (e.g., structural span; floor to ceiling height; building depth etc.); (2) capacity of positions, which evaluates the position of communication and service cores and service nets (e.g., position of stairs and elevators; position of ventilation net etc.); (3) capacity potential, which assesses the possibility for the building to be expanded or change function (e.g., loadbearing capacity of the structure; possibility to host horizontal or vertical expansions); (4) disassembly potential, which refers to the ability of the building to be easily adapted (e.g., accessibility of shaft; ease of dismantling of building elements). Seventeen rules are related to these four sub-indicators. For each rule a score between 0.1 and 0.9 is assigned. The project gets a total score based on the average of all the scores obtained rule by rule. According to the total score a building can be classified as: irreversible (building cannot accommodate any change); monofuctional (building cannot change function); transfunctional (building can be transformed from a function to another); multidimensional (building can be transformed into multiple functions). The TC is not a self-evaluated model; that means for example that a user can not award a score based on guidelines. As a result, the user must provide the project's data, while the scores are assigned by the model according to values based on literature review and case studies analysis from Dutch-based projects.

### 2.3. The application of the Reversible Design Protocol to the project of Joseph Bracops Hospital (Brussels)

The new Joseph Bracops Hospital was designed by archipelago architects (B), in partnership with NU architectuuratelier (B) and BUUR (B), who won a competition launched in 2018 by the Iris South hospitals network (Hopitaux Iris Sud—Iris Ziekenhuizen Zuid). The project of approximately 15,000 m^2^ includes the design of a new entry with a polyclinic building and a new block to host operation rooms and imaging services. Adaptability was one of the main drivers of the new design, placed in an urban tissue, surrounded by a residential neighborhood, and intended to dialog with the existing infrastructure. The project must deal with a few remains of the original hospital built at the end of the nineteenth century, progressively replaced by new buildings added without a long-term vision. The TC was applied since the preliminary design phase, during three workshops split along the design process, between RBD team and the design team. The design team transmitted the data about the project to the RBD team in a back-and-forth process aimed to orient the design choices. As soon as a change was made to the project, it was immediately evaluated by the protocol. The Joseph Bracops Hospital obtained the following scores:

• For its “capacity of dimensions” a score of 0.74; the beam and post structural system was positively assessed, as well as the regular shape of the block and the structural span of 7.5 m which allowed to properly draw the garage. Several studies have been done on the building depth in relation to typical room dimensions to test the entrance of natural light;
• For its “capacity of positions” a score of 0.82, thanks to the peripheral position of vertical circulation cores and sanitary supplies and drains and their orientation in the sense of the shorter building side;
• For its “capacity potential” a score of 0.83, because of the elevated load-bearing capacity of its structure, the surplus of space available in the shaft and the possibility to expand the volume horizontally;
• For its “disassembly potential”, the maximum score was 0.9 because of its demountable partitions and façade system and the separation between loadbearing system and services. The total score was 0.77 (on a maximum of 0.90), representing an average between all the rules of spatial and technical transformation. Buildings with a “high transformation potential” use to reach a score between 0.66-0.90, meaning that they can change function and configuration without major demolition and waste production. Actually, several functions, such as student or social housing, elderly housing, etc. were tested in the polyclinic building to check the adaptability of the plan.

## 3. Discussion

### 3.1. The current constraints of the RBD for application on healthcare buildings

The Joseph Bracops Hospital has been taken as a pilot project to test the RBD on healthcare buildings, since the model was originally developed to analyze offices, housing and schools. The designers could not intervene directly in the model, because the whole process was handled by the RBD team. This often resulted in a clash between the design and the assessment process that was not done in real time.

As highlighted in Table 1, the model only analyzes the functional and technological, which are mainly building-related, without considering the economic, social and political aspects. These latter parameters are influenced by the context and play an important role when assessing the design of a public building. Moreover, the RBD has been developed according to the Dutch building practice and market, providing feedback that could be less effective in Belgium. Besides, the model does not consider the interest in short and medium term monofunctional adaptations, which can be very important for hospitals. Monofunctional adaptation for a hospital means its capacity to adapt to changing needs, integrating strategies to optimize the secondary (partition walls, building services) and tertiary system (furniture, medical equipment etc.). Therefore, the model could integrate some parameters specific to the healthcare typologies such as: multifunctional spaces (e.g., rooms with relocatable healthcare equipment and technologies); soft spaces (e.g., spaces that surround technically sophisticated rooms helping to provide extra capacity); empty chair (e.g., leaving vacant some space for relocating functions from spaces under retrofitting) etc. (3).

### 3.2. Future research: the importance of adaptability assessment models and practitioners’ implications

Healthcare facilities are complex structures affected by social, cultural, economic and technological aspects and need to be planned and designed for present and future needs (4). In the case of healthcare architecture, the reasons for change are often better analyzed than the capacity of buildings to perform that change in practice (1). The adaptability assessment models found in the literature are oriented to assess adaptability of functions such as housing, offices, schools and generic functions or are oriented to assess the adaptability of healthcare facilities without considering their potential transformation towards other non-healthcare functions. The case study of the Joseph Bracops Hospital has been designed and tested to host a change of function with the implementation of a circular and reversible design from the level of the masterplan to the level of detailed choices of building materials (39). However, the application of the RBD protocol was in fact only applied to the polyclinic, the least complex part of the building since it hosts the outpatient clinic, and therefore with a structure already closer to offices or student housing. The case study confirms the need highlighted by the literature of data-driven and empirically validated assessment models to support the design of adaptable healthcare buildings, in order to address the questions: should hospitals give priority to monofunctional or to transfunctional/multifunctional adaptability? Should the adaptability models consider requirements related to specific functions such as healthcare? Further research will be necessary to collect data on the service life of hospitals in different geographic locations and to understand what the most frequent reason for changes or demolition are, in order to be able to draw up guidelines for a more adaptable design of healthcare buildings.

## Data Availability

The original contributions presented in the study are included in the article, further inquiries can be directed to the corresponding author.
